# Organization of atrial fibrillation using a pure sodium channel blocker: Implications of rotor ablation therapy

**DOI:** 10.1002/joa3.12844

**Published:** 2023-03-31

**Authors:** Tadafumi Nanbu, Akihiko Yotsukura, George Suzuki, Hiroyuki Takekawa, Yuki Tanaka, Katsuma Yamanashi, Masaya Tsuda, Izumi Yoshida, Masayuki Sakurai, Takashi Ashihara

**Affiliations:** ^1^ Department of Cardiovascular Medicine Hokko Memorial Hospital Sapporo‐shi Japan; ^2^ Department of Medical Informatics and Biomedical Engineering Shiga University of Medical Science Seta Tsukinowa‐cho, Otsu Japan

**Keywords:** ablation, atrial fibrillation, rotors, sodium channel blocker

## Abstract

**Background:**

Rotors are the source of atrial fibrillation (AF). However, the ablation of rotors for persistent AF is challenging. The purpose of this study was to identify the dominant rotor by accelerating the organization of AF using a sodium channel blocker and detecting the rotor's preferential area that governs AF.

**Methods:**

Overall, 30 consecutive patients with persistent AF who underwent pulmonary vein isolation and still sustained AF were enrolled. Pilsicainide 50 mg was administered. An online real‐time phase mapping system (ExTRa Mapping™) was used to identify the meandering rotors and multiple wavelets in 11 left atrial segments. The time ratio of non‐passive activation (%NP) was evaluated as the frequency of rotor activity in each segment.

**Results:**

Conduction velocity became slower—from 0.46 ± 0.14 to 0.35 ± 0.14 mm/ms (*p* = .004)—and the rotational period of the rotor was significantly prolonged—156 ± 21 to 193 ± 28 ms/cycle (*p* < .001). AF cycle length was prolonged from 169 ± 19 to 223 ± 29 ms (*p* < .001). A decrease in %NP was observed in seven segments. Additionally, 14 patients had at least one complete passive activation area. Of them, the use of high %NP area ablation resulted in atrial tachycardia and sinus rhythm in two patients each.

**Conclusions:**

A sodium channel blocker organized persistent AF. In selective patients with a wide organized area, high %NP area ablation could convert AF into atrial tachycardia or terminate AF.

## Graphical abstract

A sodium channel blocker organized persistent AF in selective paitents and non‐organized area ablation could convert AF into atrial tachycardia.

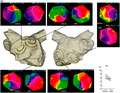

## 1 INTRODUCTION

Even in the computational mapping era,^1^ mechanism‐based ablation for persistent atrial fibrillation (AF) is challenging.^2^, ^3^, ^4^, ^5^, ^6^ Though the reason for sustained AF is not completely clear, the concept that rotors are the source of sustained AF is widely supported based on basic and clinical research in recent years.^3^, ^7^, ^8^ The self‐existing spiral wave reentry—a rotor—generates succeeding wavefronts that propagate rapidly in various directions and result in fragmentation by anatomical or functional obstacles, which generate new wavelets; of these, some result in new rotors.^9^ Therefore, detecting rotor activity, multiple wavelets activity, and distinguishing these activities from passive activity is a clue to mechanism‐based AF ablation.^7^, ^10^

In contrast, it is difficult to distinguish “dominant rotors” from “subordinate rotors” during ongoing fibrillatory conduction using a real‐time mapping system.^11^, ^12^ If a wide area of the atrium is well‐organized without fragmentation and the rotor activity is limited to a small area, the AF may be terminated by ablating the small area and, consequently, organizing AF to atrial tachycardia.^13^, ^14^, ^15^, ^16^ Furthermore, accelerating the organization of AF using a sodium channel blocker will help map them easily without considering wavefront fragmentation that becomes subordinate bystander rotors.

We hypothesized that the dominant rotor activity can be identified by accelerating the organization of AF using a sodium channel blocker and easily detecting the rotor area that governs the AF.

ExTRa Mapping, a novel online real‐time phase mapping system, is now commonly used in Japan to detect rotor activity and multiple wavelets.^17^, ^18^

## 2 MATERIALS AND METHODS

This observational prospective study was conducted between August 2020 and December 2021. We enrolled patients with persistent AF who underwent pulmonary vein (PV) isolation within 2 hours of entering the catheterization laboratory without subsequent termination of AF. All patients were refractory to drug therapies before the ablation procedure. Patients who were on amiodarone, had a history of sinus bradycardia or moderate chronic kidney disease (Creatinine >1.5 mg/dL), or were older than 85 years of age were excluded from this study. Anti‐arrhythmic drugs were discontinued before the procedure in all patients, while direct oral anticoagulants or warfarin were continued. General anesthesia and control of anti‐coagulation used in the procedures have been described previously.^19^

Before starting this study, we performed rotor ablation as additional ablation for paroxysmal and persistent AF patients using ExTRa Mapping between Aug 2019 and July 2020. During this period, 20 patients exhibited persistent AF that was resistant to repetitive ablation. The outcome of these 20 patients is shown Supporting Information Figure S1. In this preliminary observation, most patients (14/20) had already undergone pulmonary vein isolation (PVI) and roofline ablation by cryoballoon or PVI and posterior wall isolation by radiofrequency (RF) ablation. We used these preliminary observation groups as the control group to confirm the clinical impact of this study.

### 2.1 Ablation procedure

A 20‐pole catheter (BeeAT, Japan Lifeline Shinagawa, Tokyo Japan) was inserted via the internal jugular vein and positioned into the distal coronary sinus along the right atrial lateral wall.

A single transseptal puncture was performed using a RF‐powered transseptal needle (RF needle, Baylis Medical Inc., Montreal, Quebec, Canada) using an 8‐Fr sheath (SL0, St. Jude Medical, MN, USA) under 10‐Fr intracardiac echo guidance (ViewFlex Xtra, Abbott, MN, USA). After insertion of the 8‐Fr sheath into the left atrium (LA), it was exchanged for an 8.5‐Fr deflectable sheath (Agilis™ NxT, Abbott) over the guidewire. NavX mapping system (EnSite Precision™, Abbott) or CARTO mapping system (CARTO® 3, Biosense Webster, Diamond Bar, CA, USA) were used to obtain the voltage map and RF ablation.

After an electro‐anatomical map during the AF was obtained, extensive encircling PV isolation was performed according to indexed ablation. On the NavX system, RF energy was delivered using a 4‐mm irrigated‐tip catheter (TactiCath™ SE, Abbott) with the following parameters: energy, 35 W; contact force, 5–20 g; targeting lesion size index, 4.5–5. On the CARTO system, using a similar 4‐mm irrigated‐tip catheter (THERMOCOOL SMARTTOUCH™ SF, Biosense Webster), the parameters were the following: energy, 40 W; contact force, 5–20 g; ablation index, 400–450. Wide area ablation was confirmed based on the elimination of bipolar signals on the voltage map (<0.1 mV) or the detection of a dissociated signal from the isolated area.

### 2.2 Electrophysiological study

#### 2.2.1 ExTRa mapping

**FIGURE 1:**
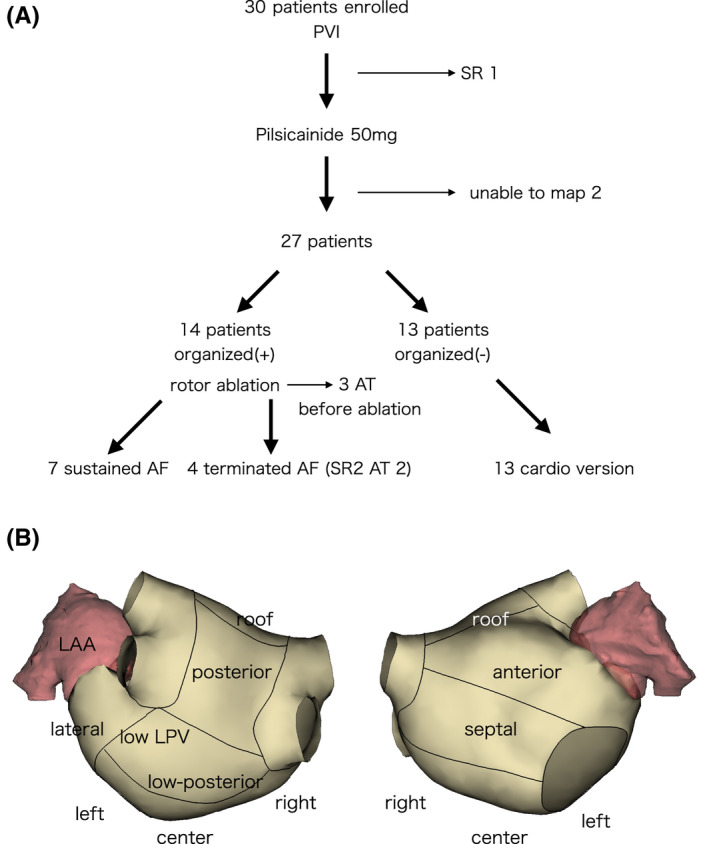
Schematic flowchart of the study protocol (upper panel) and segmentation of the left atrial surface into 11 parts (lower panel). center, center inferior area; LAA, left atrial appendage; left, left inferior area; low LPV, lower left pulmonary vein; Right, right inferior area.

ExTRa Mapping™ is an online real‐time phase mapping system,^17^ which is classified as contact mapping and is well‐validated in optical mapping.^18^ In brief, 32 real bipolar signals and nine virtual bipolar signals were recorded using a 20‐pole spiral‐shaped deflectable catheter (Reflexion HD; Abbott). The concept of this phase map is based on the restitution property of human atrial action potentials during non‐paroxysmal AF. It is closely related to the preceding diastolic interval,^20^, ^21^ and we can chart a phase map from the local bipolar signals. Based on this theory, action potentials were simulated from bipolar signal timings,^17^, ^18^ and 5‐s spatio‐temporal phase map (approximate region of interest, 2.5 cm in diameter; 4 ms per frame) was automatically created using wavefronts and phase singularities. The shadow of Reflexion HD was projected on the left atrial electro‐anatomical map, and the acquisition of signal was obtained from each segment (roof, posterior, lower posterior, lower left PV, left lateral, left bottom, center bottom, right bottom, anterior, septum, and left atrial appendage [LAA]) (Figure 1B). The catheter was moved to each segment so that most of the real bipolar signals exceeded 0.05 mV. After the catheter was stabilized, the shadow of the catheter was confirmed in 3‐dimensional geometry. More than 5 areas were sampled in each patient. According to the number of phase singularities, meandering rotors, which are defined as within 2 phase singularities, and multiple wavelets, defined as more than 2 phase singularities, were automatically classified. The periods of meandering rotors and multiple wavelets were summed respectively and indicated on the phase map. These meandering rotors and multiple wavelets were defined as non‐passive activation. The value of “non‐passively activated ratio” (%NP) was automatically calculated as the ratio of the non‐passive activation period to the recording time.

#### 2.2.2 Reproducibility of ExTRa mapping

Rotors usually meander, and phase singularities drift out of the region of interest (i.e., out of the spiral catheter) within a short period. Before the present study, we repeated the measurement of %NP 10 times in 6 patients in various areas (Supporting Information Table S1). In this series, a Shapiro–Wilk test indicated that most data were consistent with a normal distribution and that the moving average for the 15 consecutive seconds was enough to represent the mean %NP value. Therefore, we repeated the measurement of %NP thrice (15 s) in each area and used the mean value. In this series, no area showed %NP 0% repetitively. In contrast, atrial tachycardia always shows 0% %NP because all waves are planar (not shown). Thus, we defined newly developed repetitive 0% % NP as AF organization.

#### 2.2.3 Administration of pilsicainide and measurement of electrophysiological data

**FIGURE 2:**
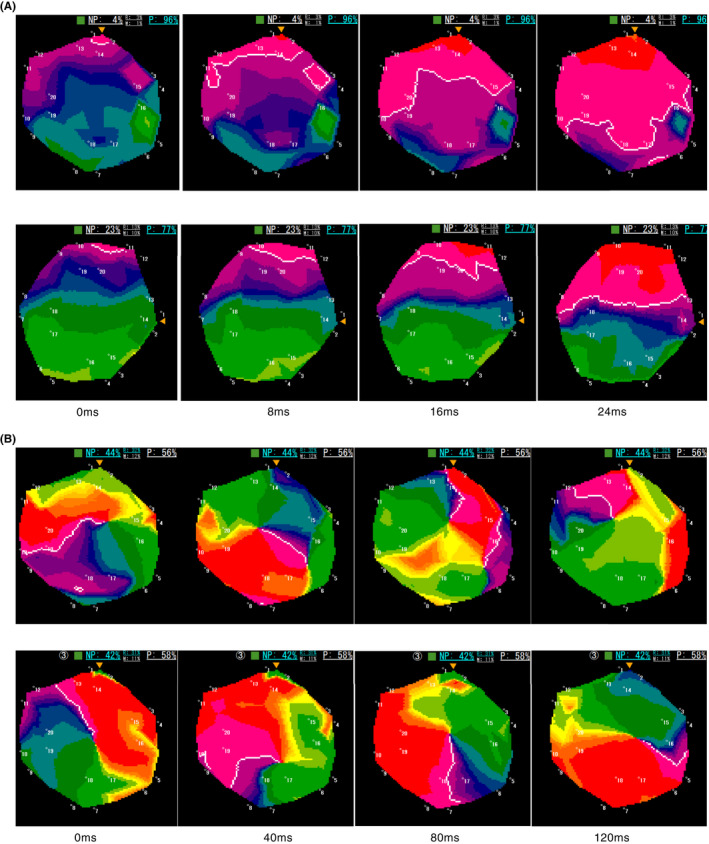
(A) A phase map recorded in the left atrial roof before and after pilsicainide administration (upper two panels). The white line shows the wavefront. Emergence of wavefront was set at 0 ms. The wavefront propagated almost all the areas 24 ms later. After pilsicainide administration, the wavefront reached only half the area due to reduced conduction velocity. (B) A phase map recorded in the left atrial posterior wall in another patient before and after (lower two panel) pilsicainide administration. Before pilsicainide, one rotation was almost complete 120 ms later. After pilsicainide, the rotation remained semicircular in the same period.

Pilsicainide was administered at a rate of 25 mg/min for a total dose of 50 mg over 2 min. Electrophysiological data were collected before and 5 min after pilsicainide was administered. The mean duration of the AF cycle was calculated from the coronary sinus electrogram using manual annotation for 10 consecutive signals. The conduction velocity (CV) during AF was measured on the maps. CV is greatly influenced by the wavefront curvature, that is, as the curvature becomes steep, the CV decreases. We chose a low %NP area both before and after pilsicainide administration in an identical area and selected a planar wavefront that propagated from one edge to the opposite edge with the least rotation and defined this duration required as the wave crossing time. CV was calculated as the diameter of the catheter divided by the wave crossing time (Figure 2A).

A rotor is a spiral wave of excitation that rotates around a phase singularity for one or more cycles.^9^, ^14^ The rotational period was also determined on the maps (Figure 2B). In each patient, two identical areas with relatively high %NP were chosen and phase singularity meandering near the center of the region of interest was carefully selected for evaluation. The period of wavefront rotation around the phase singularity (i.e., rotor) in one cycle was calculated by the frame count.

AF organization is characterized by a reduced number of wavefronts in comparison with the original AF and is detected as reduced wavelets or decreased phase singularities. If %NP approaches 0% area *after* pilsicainide administration, we classified the case in the AF organization group (Figure 1A).

The dominant frequency (DF) was obtained at the same time in the same area of ExTRa mapping using Reflexion HD catheter. All signals were collected for 5 s at a sampling rate of 1000 Hz and analyzed using software embedded in the polygraph (RMC 5000 Nihon Kohden Co. Tokyo, Japan). Thirty‐two points of DF values and regularity indices were shown simultaneously at one area, and the highest DF value with a regularity index ≥0.2 represented the DF value of the area. The DF gradient of each patient was defined as the maximal value–minimal value of the highest DF among 11 areas.

#### 2.2.4 Rotor ablation in organized AF

**FIGURE 3:**
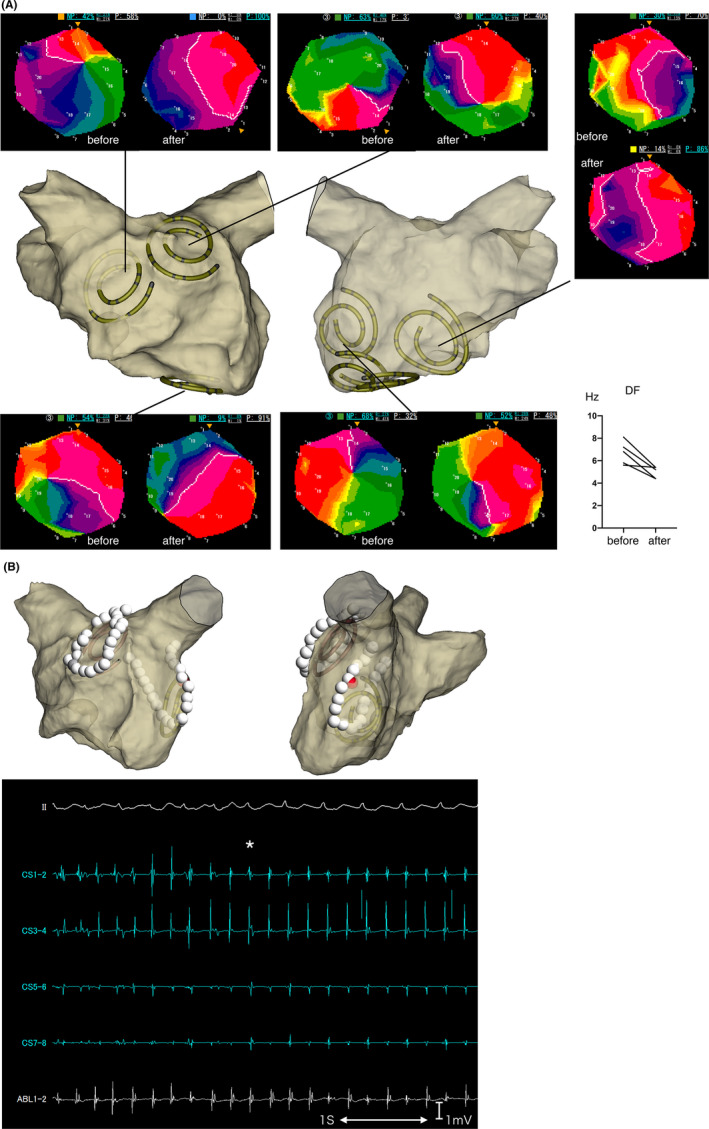
(A) A 65‐year‐old male patient with wide area organization before and after pilsicainide administration. Left pulmonary vein is the common trunk. %NP value of septal, inferior, and lower posterior areas reduced drastically after pilsicainide administration, while those of anterior and lateral sites remained high. (B) Ablation point of the same patient and intracardiac electrogram. Two high %NP areas were targeted. During the lateral site encircling ablation, atrial fibrillation was converted into atrial tachycardia (asterisk). Red dot indicates the converted point. CS, coronary sinus; DF, dominant frequency. CS1‐2 is located in the distal CS and CS7‐8 is located in the proximal CS.

**FIGURE 4:**
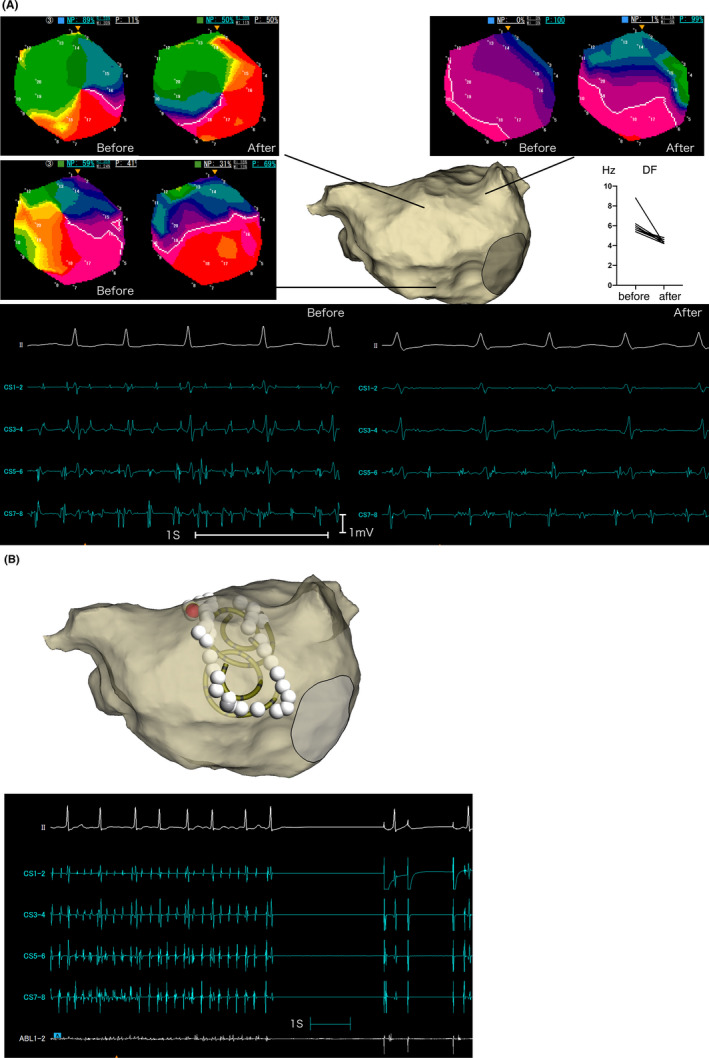
Persistent atrial fibrillation that lasted for 3 years in a 63‐year‐old female patient. (A) Phase maps were recorded in the anterior wall, left atrial appendage (LAA), and center of the inferior wall before and after pilsicainide administration. Anterior wall phase map showed rotational activity (upper left) and LAA (upper right) showed passive activity. Both excitation patterns were unchanged after pilsicainide administration. In contrast, the rotational activity disappeared in the inferior wall phase map (middle, right, and left panel) after pilsicainide administration. Lower panel: surface electrocardiogram (II lead) and intracardiac electrogram before (lower left) and after (lower right) pilsicainide administration. Atrial fibrillation cycle length was prolonged and the local signal was decreased and became less fragmented after pilsicainide administration. DF, dominant frequency; CS, coronary sinus. CS1‐2 is the distal site and CS7‐8 is a proximal site. (B) Ablation site (upper panel) and intracardiac electrogram at atrial fibrillation (AF) termination (lower panel). Linear ablation (white dot) was performed along the high %NP area. Red dot shows termination point of AF. Please note that irregular and fragmented electrogram changed into a regular discrete one, the AF cycle length became prolonged, and finally converted into sinus rhythm. CS, coronary sinus. CS1‐2 is the distal site and CS7‐8 is the proximal site.

If the rotational activity is observed only in one or two areas and other large areas are low %NP areas, the rotational activity area (high %NP area) is regarded as the source of AF controlling the other areas.^13^ Therefore, we performed a high %NP area ablation in only the organized group (Figure 1A). We defined %NP > 50% as high %NP area.^17^ Ablation was performed point‐by‐point to surround the high %NP area. Power setting, targeting ablation index, or lesion size index were the same as the PVI (see Ablation Procedure). If the high %NP area was close to unexcitable areas, such as the mitral annulus, a large scar, or a previous ablation line for PV isolation, we connected the ablation point to the unexcitable area (Figures 3 and 4, Supporting Information Figures S2 and S3) to prevent iatrogenic atrial tachycardia. The endpoint of ablation was encircling the NP area by indexed ablation.

### 2.3 Study follow‐up

Follow‐up appointments were scheduled at 1, 3, and 6 months after the procedure. At each visit, we collected data regarding the medical history and performed a detailed physical examination including a 12‐lead electrocardiogram (ECG). During the follow‐up, the patients were free to visit our hospital at any time in case of arrhythmia symptoms, at which time a 12‐lead ECG was recorded. A Holter ECG was obtained at 6 months post‐procedure or at other time points during symptoms suggestive of a recurrence of arrhythmia.

### 2.4 Statistical analysis

Data are expressed as mean ± standard deviation or median (25th–75th percentile). Comparisons between intra‐patient data were performed using the Wilcoxon signed‐rank test or *χ* ^2^ analysis. Comparisons between patient groups were performed using the Kruskal–Wallis test. Arrhythmia‐free survival curves for each group are presented as Kaplan–Meier plots, and time‐to‐event analysis was performed using the log‐rank test. Dates were censored in April 2022. Patients who were lost to follow‐up were censored at the date of the last visit.

## 3 RESULTS

### 3.1 Patient characteristics and procedural results

We recruited 30 consecutive patients in the present study. The baseline patient characteristics are summarized in Table 1. Three patients were excluded from the analysis due to termination of AF after PV isolation (*n* = 1) and attenuation of local signal (<0.05 mV) after pilsicainide administration and unreliable ExTRa Mapping data (*n* = 2). Fourteen patients had approximately 0% %NP at least in one area and were classified in the organized group. Thirteen patients showed no organization (sustained group). One of the patients in the sustained group developed transient atrial tachycardia but degenerated into AF in a short period. In the sustained group, sinus rhythm was achieved in all patients using cardioversion at the end of the procedure, and no ExTRa Mapping‐guided ablation was performed in this group.

**TABLE 1:** Basic patient characteristics.

	Sustained group	Organized group	Preliminary control	*p*
Patients	13	14	20	
Male gender *n*, (%)	18 (90)	5 (71)	14 (70)	.32
Age, years	67.8 ± 21.0	70.3 ± 4.4	66.1 ± 11.0	.13
time to diagnosed, years	4.3 ± 4.0	4.3 ± 3.5	5.6 ± 4.4	.77
Tried AADs	1.6 ± 1.0	1.4 ± 0.9	2.1 ± 1.1	.14
Height, cm	168.0 ± 9.3	166.3 ± 14.4	164.0 ± 8.4	.29
Weight, kg	72.5 ± 12.9	63.0 ± 16.0	67.7 ± 13.5	.08
BMI	25.5 ± 3.0	22.4 ± 2.9	24.9 ± 3.7	.08
Hypertension	11 (55)	7 (50)	10 (50)	.79
Diabetes	4 (20)	1 (14)	2 (10)	.67
CHF	15 (75)	5 (71)	13 (65)	.77
Prior stroke	6 (30)	1 (14)	3	.64
Ischemic heart disease	0 (0)	0 (0)	2 (10)	.25
Valvular heart disease	1 (5)	0 (0)	0	.28
CKD	2 (10)	2 (29)	5 (25)	.67
LVEF (%)	55.2 ± 15.1	60.7 ± 9.2	60.8 ± 9.7	.04
LA diameter, mm	47.0 ± 5.8	38.7 ± 8.2	40.7 ± 5.3	<.001
LA volume, mL	211 ± 61	168 ± 37	179 ± 36	.09
rotor ablation (%)	0 (0)	11 (78)	20 (100)	<.01
Prior PV ablation	1 (8)	6 (42)	17 (85)	<.001

Abbreviations: AADs, anti‐arrhythmics; BMI, body mass index; CHF, congestive heart failure; CKD, chronic kidney disease; LA, left atrium; LVEF, left ventricular ejection fraction; PV, pulmonary vein.

### 3.2 Effect of pilsicainide on persistent AF

The effects of pilsicainide on all persistent AF are summarized in Table 2. The AF cycle length was prolonged from 169 ± 18 to 223 ± 29 ms (*p* < .001). The CV slowed from 0.46 ± 0.13 to 0.35 ± 0.15 mm/ms (*p* = .004), The rotational period of the rotor was prolonged significantly from 156 ± 21 to 192 ± 28 ms/cycle (*p* < .001). There was no area with an increase in the %NP. The %NP derived from the meandering rotors decreased significantly in 3/11 areas, whereas the %NP derived from the multiple wavelets decreased in 9/11 areas. Total %NP value decreased in 7/11 areas, and most of them (6/7) were consistent with the decreased area as per the multiple wavelets.

**TABLE 2:** Electrophysiological data in all patients.

	Control	Pilsicainide	*p*
AF cycle length (ms)	169 ± 18	223 ± 29	<.001
Conduction velocity (mm/ms)	0.46 ± 0.13	0.35 ± 0.15	.004
Rotational period (ms/cycle)	156 ± 21	192 ± 28	<.001
High NP area (*n*)	1.6 ± 1.6	0.9 ± 1.0	.029
%*NP value*			
Roof (*n* = 17)			
Total	32 ± 18	22 ± 16	.125
Meandering rotors	18 ± 10	14 ± 10	.209
Multiple wavelets	14 ± 10	8 ± 6	.049
Posterior (*n* = 14)			
Total	35 ± 15	25 ± 19	.035
Meandering rotors	18 ± 8	17 ± 13	.363
Multiple wavelets	17 ± 9	8 ± 7	.002
Lower posterior (*n* = 24)			
Total	33 ± 12	21 ± 20	.008
Meandering rotors	20 ± 8	14 ± 14	.039
Multiple wavelets	13 ± 7	7 ± 8	.003
Lower left PV (*n* = 8)			
Total	29 ± 17	18 ± 19	.036
Meandering rotors	17 ± 8	11 ± 14	.123
Multiple wavelets	12 ± 10	7 ± 7	.051
Center inferior (*n* = 25)			
Total	39 ± 16	29 ± 19	.033
Meandering rotors	24 ± 11	18 ± 12	.106
Multiple wavelets	16 ± 8	11 ± 8	.027
Left inferior (*n* = 23)			
Total	41 ± 19	29 ± 14	.031
Meandering rotors	24 ± 12	17 ± 10	.018
Multiple wavelets	16 ± 10	12 ± 8	.162
Right inferior (*n* = 20)			
Total	32 ± 16	26 ± 22	.046
Meandering rotors	18 ± 9	17 ± 16	.480
Multiple wavelets	14 ± 10	9 ± 7	.001
Lateral (*n* = 12)			
Total	28 ± 20	20 ± 22	.239
Meandering rotors	16 ± 11	13 ± 16	.265
Multiple wavelets	12 ± 11	7 ± 8	.033
Left atrial appendage (*n* = 22)			
Total	26 ± 21	20 ± 21	.136
Meandering rotors	15 ± 12	13 ± 14	.409
Multiple wavelets	11 ± 10	7 ± 8	.049
Anterior (*n* = 26)			
Total	41 ± 18	34 ± 20	.069
Meandering rotors	24 ± 10	24 ± 17	.492
Multiple wavelets	17 ± 11	10 ± 6	.006
Septal (*n* = 22)			
Total	34 ± 19	26 ± 22	.009
Meandering rotors	20 ± 11	15 ± 10	.042
Multiple wavelets	14 ± 9	8 ± 6	.002

Abbreviation: AF, atrial fibrillation.

The mapping data of the organized and sustained groups are summarized in Table 3. At baseline, the AF cycle length, CV, and rotational period were not different between the groups. The AF cycle length was prolonged significantly in both groups (sustained group, 172 ± 22 to 215 ± 26 ms; organized group, 166 ± 14 to 229 ± 31 ms). The CV in the organized group decreased significantly with pilsicainide (0.47 ± 0.15 to 0.31 ± 0.08 mm/ms; p = 0.006); however, the change was not significant in the sustained group (0.45 ± 0.11 to 0.40 ± 0.19 mm/ms). The rotational period of rotors was significantly prolonged in both groups (sustained group, 158 ± 26 to 191 ± 27 ms/cycle; organized group, 153 ± 13 to 190 ± 29 ms/cycle). There was no difference in the AFCL and the rotational period *after* the administration of pilsicainide between the groups. CV was significantly slower in the organized group than that in the sustained group *after* pilsicainide.

**TABLE 3:** Electrophysiological change with pilsicainide.

	Organized (*n* = 14)	Sustained (*n* = 13)
AF cycle length (ms)		
CTL	166 ± 14	172 ± 22
Pil	229 ± 31	215 ± 26
Conduction velocity (mm/ms)		
CTL	0.47 ± 0.15	0.45 ± 0.11
Pil	0.31 ± 0.08	0.40 ± 0.19
Rotational period (ms/cycle)		
CTL	190 ± 29	195 ± 27
Pil	153 ± 13	158 ± 26
Number of high NP area		
CTL	1.3 ± 1.2	2.1 ± 1.9
Pil	1.1 ± 1.1	0.6 ± 1.0
%*NP value of each area*		
Roof		
CTL	26 ± 18	36 ± 17
Pil	17 ± 16	25 ± 16
*p* value (vs. CTL)	.50	.17
Posterior		
CTL	24 ± 16	41 ± 10
Pil	13 ± 14	31 ± 19
*p* value (vs. CTL)	.14	.11
Lower posterior		
CTL	33 ± 14	34 ± 11
Pil	17 ± 23	26 ± 13
*p* value (vs CTL)	.02	.25
Lower left PV		
CTL	20 ± 13	32 ± 21
Pil	17 ± 24	20 ± 16
*p* value (vs. CTL)	.27	.07
Center inferior		
CTL	42 ± 19	37 ± 12
Pil	19 ± 17	39 ± 16
*p* value (vs. CTL)	.007	.97
Left inferior		
CTL	45 ± 21	37 ± 14
Pil	29 ± 13	29 ± 20
*p* value (vs. CTL)	.08	.24
Right inferior		
CTL	31 ± 19	35 ± 15
Pil	23 ± 30	26 ± 11
*p* value (vs. CTL)	.40	.08
Lateral		
CTL	31 ± 17	24 ± 26
Pil	23 ± 24	14 ± 16
*p* value (vs. CTL)	.26	1.00
Left atrial appendage		
CTL	20 ± 24	30 ± 18
Pil	9 ± 17	28 ± 20
*p* value (vs. CTL)	.04	.64
Anterior		
CTL	39 ± 19	44 ± 16
Rotor	35 ± 23	33 ± 16
*p* value (vs. CTL)	.38	.08
Septal		
CTL	23 ± 15	45 ± 16
Pil	22 ± 18	24 ± 12
*p* value (vs. CTL)	.56	.003

Abbreviations: AF, atrial fibrillation; CTL, control; Pil, pilsicainide.

At baseline, there was no difference in total %NP between the groups in all areas. After pilsicainide administration, the total %NP value significantly decreased in 3 areas (lower posterior wall [33 ± 14 to 17 ± 23; *p* = .019], center of inferior wall [42 ± 19 to 19 ± 17; *p* = .007], and LAA [20 ± 24 to 9 ± 17; *p* = .04]) in the organized group. In contrast, in the sustained group, the %NP of the center inferior area (37 ± 12 to 39 ± 16; *p* = .97) and the LAA (30 ± 18 to 28 ± 20; *p* = .64) were not sensitive to pilsicainide. Moreover, in the [A12] sustained group, a decrease in total %NP value was observed in only one area (septum, 45 ± 16 to 24 ± 12; *p* = .003).

### 3.3 Change in high %NP

High %NP areas were observed in 19 patients (70%, not shown in the table). Of these, the high %NP areas changed in 5 patients and remained unchanged in 14 patients after pilsicainide administration. The number of high %NP areas decreased from 1.6 ± 1.6 to 0.9 ± 1.0 (*p* = .029) (Table 2).

#### 3.3.1 Change in highest DF

The highest DF in each area at baseline was not significantly different between the organized group and sustained group except for the septal area. The highest DF markedly decreased in every area after pilsicainide administration in both groups (Supporting Information Table S2).

At baseline, the DF gradient was slightly higher in the organized group than in the sustained group (1.1 ± 0.9 Hz, vs. 0.9 ± 0.4 Hz *p* = .07) but the difference became non‐significant between two groups (organized 1.0 ± 0.9 Hz, sustained 0.9 ± 0.2 Hz *p* = .15). In this study, 11 patients received rotor ablation. The ablation area (which is a high %NP area after pilsicainide administration) was coincident with the highest DF area in three patients (3/11, 27%) before pilsicainnide administration and in three patients (3/11, 27%) after pilsicainide administration.

### 3.4 Outcome of ExTRa mapping‐guided ablation

In 3 patients in the organized group, AF organized into atrial tachycardia before rotor ablation. Atrial tachycardia was confirmed by regular intracardiac signals and a %NP value of approximately 0% in any area. In these patients, sinus rhythm was achieved using linear ablation. The remaining 11 patients received rotor ablation. In 2 patients, AF organized to atrial tachycardia after rotor ablation (Figure 3), which was mapped by local activation timing and returned to sinus rhythm using linear ablation. In two patients, AF returned to sinus rhythm after rotor ablation (Figure 4). Therefore, sinus rhythm was returned in 4/14 (29%) patients in the organized group using ExTRa Mapping‐guided catheter ablation. Overall termination of AF or atrial tachycardia using catheter ablation was achieved in seven patients in the organized group.

The number of organized areas in these seven patients was significantly higher than that in the non‐termination patients (4.9 ± 2.6 areas vs. 1.0 ± 1.2 areas, *p* < .01).

**FIGURE 5:**
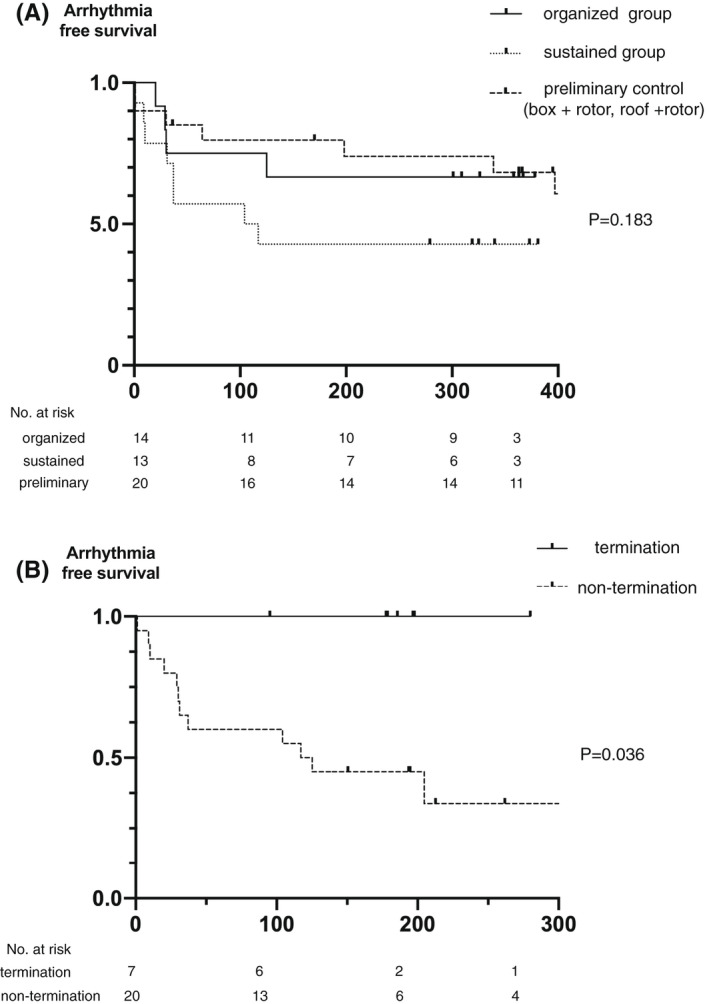
Kaplan–Meier curve of arrhythmia‐free survival. (A) among the organized group, sustained group, and preliminary control. (B) between atrial fibrillation termination group and non‐termination group.

Recurrence of atrial arrhythmia beyond the 1‐month blanking period was observed in 3 patients in the organized group and 7 patients in the sustained group. In the organized group, all 3 patients developed recurrence of AF. In the sustained group, 2 patients developed recurrence as atrial tachycardia and 5 patients developed AF. Kaplan–Meier estimates of 9‐month arrhythmia‐free survival after the procedure was 69.2% in the organized group, 42.8% in the sustained group, and 70.0% in the preliminary control (log‐rank chi‐squared 3.39; *p* = .18; Figure 5A). To confirm the effects of the termination of AF or atrial tachycardia using ablation, we compared the groups with and without AF termination. The 9‐month arrhythmia‐free survival was 100% in the group with termination and 49.9% in the group without termination (log‐rank chi‐squared, 4.38, *p* = .04; Figure 5B).

## 4 DISCUSSION

This is the first study to evaluate the effects of a pure sodium channel blocker using a contact phase map in human persistent AF. The major finding of this study is that pilsicainide decreased the CV, slowed the rotational period of rotors and prolonged the AF cycle length. Another finding is that organization of AF into atrial tachycardia (or atrial flutter) is closely related to a reduction in rotor activity in several areas. Lastly, ablation of high rotor activity observed after pilsicainide can organize AF into atrial tachycardia or sinus rhythm in some cases.

In an induced canine AF model, pilsicainide decreased CV and increased the effective refractory period.^22^, ^23^, ^24^ Although we could not measure the refractory period, CV during AF has been reported to be decreased in vitro human atrium with AF.^25^ In most animal AF models, the longevity of persistence is extremely shorter than that in human persistent AF, and it is uncertain if their findings apply to long‐lasting human AF. These results demonstrate that pharmacological effects can be achieved in human persistent AF with a clinical dose. Generally, an enlarged atrium includes structural remodeling because of fibrosis and electrical remodeling with various types of ionic current abnormalities.^26^, ^27^ Prolongation of AF cycle length and slowing of the rotational period observed in our series also suggest that the use of sodium channel blockers is a potential pharmacological intervention in persistent AF. However, AF cycle length or rotational period alone cannot become a surrogate for the organization of AF.

In this study, pilsicainide significantly reduced multiple wavelets in several areas. On the ExTRa Mapping system, wave dynamics manifesting more than 2 phase singularities in a region of interest is classified as multiple wavelets. The reduction of multiple wavelets implies that less phase singularities are observed in a region of interest. Theoretically, the rotor will continue to rotate even if the CV or excitability is reduced. Two reasons have been postulated for the decreased number of phase singularities in a region of interest. One is that the rotor core size becomes large and the rotor (with phase singularity) meanders out of the region of interest. Pilsicainide is reported to make the original rotor circuit size large and slow the rotational period.^14^, ^22^ If the tissue excitability decreases, a steep curvature of the wave will not excite the adjacent tissue and the ability to propagate is consequently decreased; therefore, the wave tip should rotate around a large core.^15^ The second postulated mechanism is that the generation of a new rotor is inhibited by pilsicainide. Pilsicainide shortens the wavelength and widens the excitable gap.^23^ Consequently, the wavefront becomes difficult to meet the wave tail and the wavefront‐wavetail interaction will occur less.

In our series, the termination of AF using rotor ablation was still challenging. We could terminate AF in 50% (7 /14) of patients in the organized group. However, the organization was pharmacologically achieved in three patients, and we could terminate AF using ablation in only four (28%) patients. This success rate suggests that dominant rotor theory is not necessarily true with regard to persistent AF. The theory that the abolishing dominant rotor can terminate AF applies to cases wherein the rotor is fixed or meanders a narrow area such as the pulmonary vein or superior vena cava. In this study, 13 of 27 patients failed to exhibit organized AF, which suggests that several rotors existed and interacted; accordingly, it was difficult to determine the dominant rotor in persistent AF.

The concept of our rotor ablation is to change the behavior of the rotor by anchoring it to an unexcitable region (i.e., ablation region) and convert it to atrial tachycardia or eliminate patient‐specific AF sources (AF‐sustaining substrates).^28^, ^29^, ^30^, ^31^ In our data, stable atrial tachycardia was achieved in 2 patients. Two patients who developed sinus rhythm demonstrated transient organization into atrial tachycardia before termination. On the ExTRa Mapping system, the region of interest was a narrow circular area (approximately 2.5 cm in diameter) and we could not directly visualize the rotor anchoring to the ablation lesion. However, the conversion to atrial tachycardia in all 4 patients supports the concept of anchoring indirectly.

A high %NP indicates a high risk of rotors, i.e., frequent passage of meandering rotors or high probability of new meandering rotors and/or multiple wavelets in the region of interest. At baseline, %NP was similar at several sites, and we could not identify the appropriate ablation sites. Although we can arrange %NP of different areas and ablate them accordingly, not all rotors are effective target sites.^17^ The wavefront‐wavetail interactions are common with an increasing number of rotors. All rotors meander, and the wavefronts will cause new wavefront‐wavetail interactions elsewhere; subsequently, new rotors keep developing. Therefore, all left atrial areas should be targeted as the ablation site, and the %NP area ablation may not be practical. After administration of pilsicainide, a high %NP area remerged in the organized group. It is ideal to ablate the rotor area that governs other large areas^13^, ^32^; however, such an ideal situation was rarely observed in our series. In intention to treat analysis, the 9‐month arrhythmia‐free rate was higher in the organized group, but the difference was not statistically significant between the groups. In contrast, once termination was achieved, most patients developed no recurrence of atrial tachyarrhythmia. Regular atrial tachycardia is sometimes observed after PV isolation, some of which are macro‐reentry tachycardia.^33^ Treating pilsicainide‐induced atrial tachycardia may mimic atrial tachycardia following PV isolation.

The clinical benefit of administering additional pilsicainide is that it aids in determining, which patients are likely to change to AT from persistent AF or to organize AF into AT with a limited ablation area, which leads to termination of arrhythmia. In contrast, the use of anti‐arrhythmics may mask the non‐PV foci. A pilsicainide challenge might be useful, especially in patients who could not be induced or in whom it is difficult to determine non‐PV foci.

The DF gradient was slightly higher in the organized group. Some patients in the organized group showed a high DF area before pilsicainide administration (Figure 4), which implies that a dominant rotor might exist in some cases in the organized group. However, the ablation area did not always correlate with the highest %NP area. Since the rotor meanders, the location of the highest NP area may vary. Accordingly, both indices should be used complementarily to reveal the AF maintenance mechanism.^34^

### 4.1 Study limitations

This study was a single‐center retrospective observational analysis and included a small number of patients. Furthermore, the %NP value after rotor ablation was not clear. We tried to confirm the activity after encircling the NP area; however, reliable ExTRa Mapping was often difficult due to low signal amplitude. It was difficult to map all the left atrial areas in patients with a small atrium. Some electrodes of the spiral catheter did not contact the tissue sufficient enough to record the local signal or the catheter deformed in the atrium, especially in the low septal area and right inferior area. Additionally, buprenorphine can inhibit the sodium current, and propofol can inhibit sodium, calcium, and potassium currents and may interact with sodium channel blockers, although the concentrations needed for inhibition is much higher than concentrations that are clinically achievable.^35^ Finally, low‐voltage areas were not determined during sinus rhythm at baseline; thus, the role of low‐voltage areas in rotor initiation or maintenance is unclear.

## 5 CONCLUSIONS

We investigated the effects of a sodium channel blocker in persistent AF. The reduction of the rotors was closely related to the organization of AF. Intensive organization is achieved in 50% of cases at a clinical dose. Instantaneous termination of AF using rotor ablation was achieved in only 28% of patients with well‐organized AF. However, termination of AF resulted in improved outcomes.

## AUTHOR CONTRIBUTIONS

*Concept/design*: Tadafumi Nanbu. *Data analysis/interpretation*: Tadafumi Nanbu, Akihiko Yotsukura. *Drafting article*: Tadafumi Nanbu. *Critical revision of article*: Takashi Ashihara. *Approval of article*: Izumi Yoshida, Masayuki Sakurai. *Data collection*: George Suzuki, Hiroyuki Takekawa, Yuki Tanaka, Katsuma Yamanashi, Masaya Tsuda.

## CONFLICT OF INTEREST STATEMENT

The authors declare no conflict of interests for this article.

## ETHICS APPROVAL

The study protocol was approved by the institutional review board of our hospital.

## PATIENT CONSENT

Patient consent was obtained with written form and consent for inclusion was obtained by an opt‐out method.

## Supporting information


Supplementary Figure 1.

Supplementary Figure 2.

Supplementary Figure 3.

Supplementary Table 1.

Supplementary Table 2.

## Data Availability

The data that support the findings of this study are available from the corresponding author (Tadafumi Nanbu), upon reasonable request.
