# Supplementary figures and images for: Organization of atrial fibrillation using a pure sodium channel blocker: Implications of rotor ablation therapy
Source: J Arrhythm. 2023 Mar 31;39(3):327–40. doi: 10.1002/joa3.12844 (PMC10264751; doi:10.1002/joa3.12844)

**Arrhythmia  
free survival**

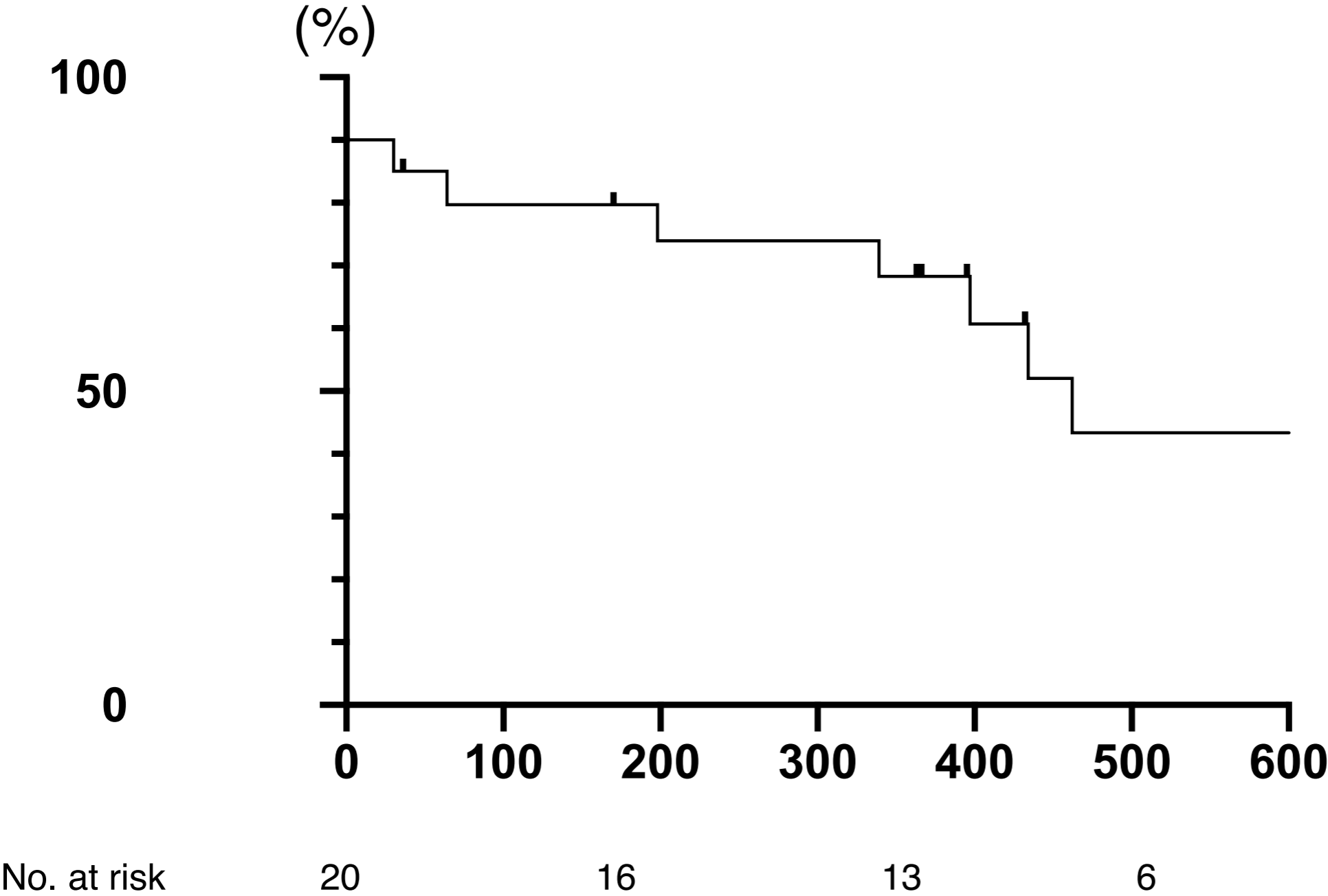

Supplement: Supplementary file 1 — Supplementary Figure 1. [file JOA3-39-327-s003.pdf]

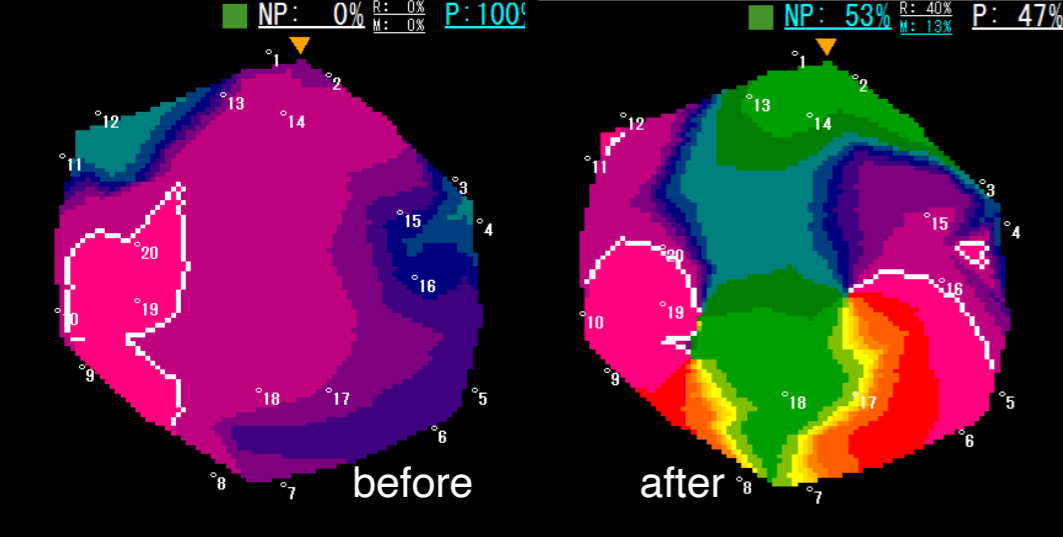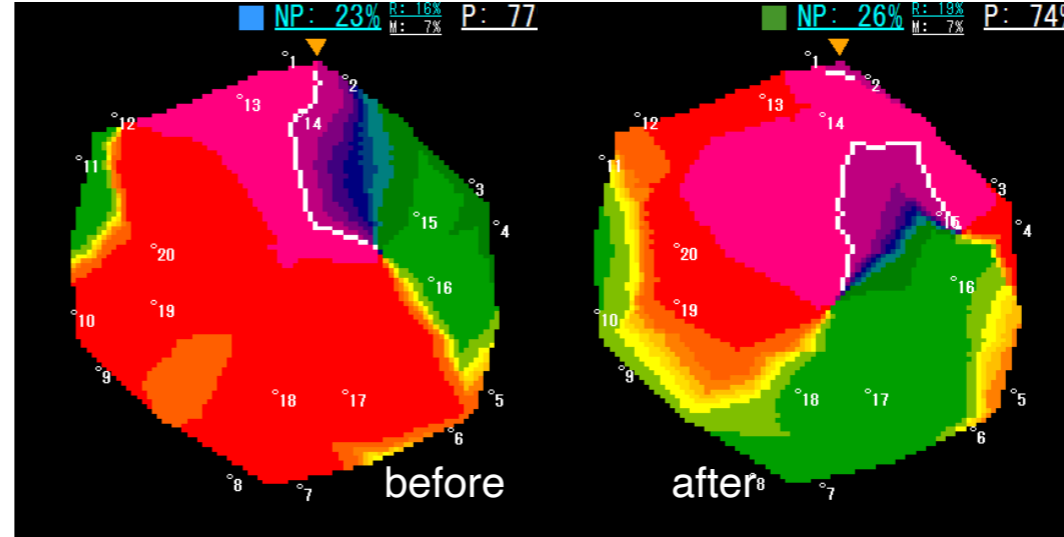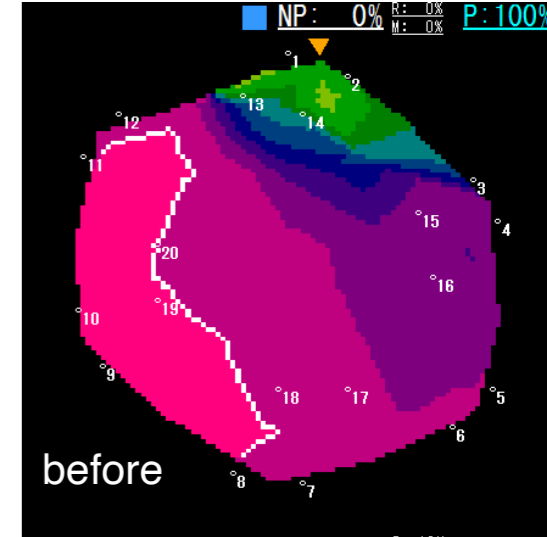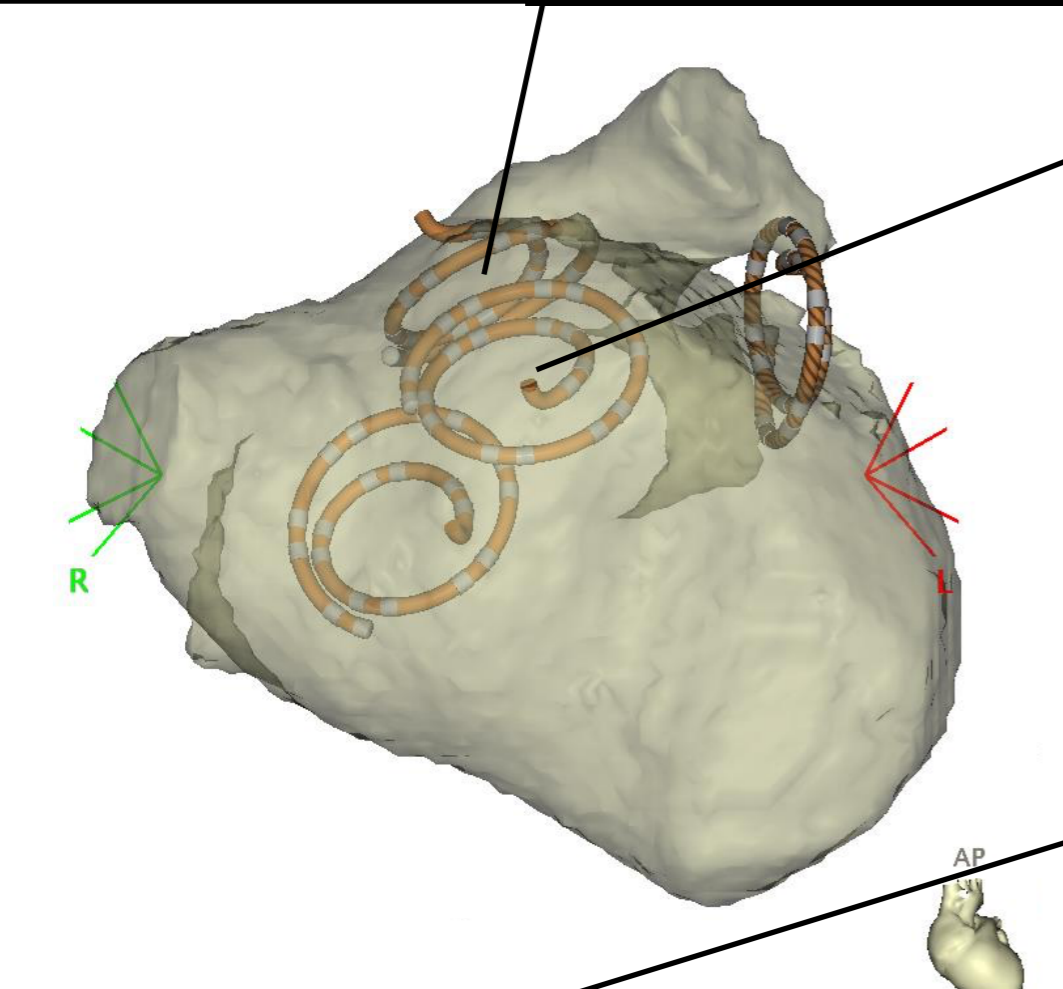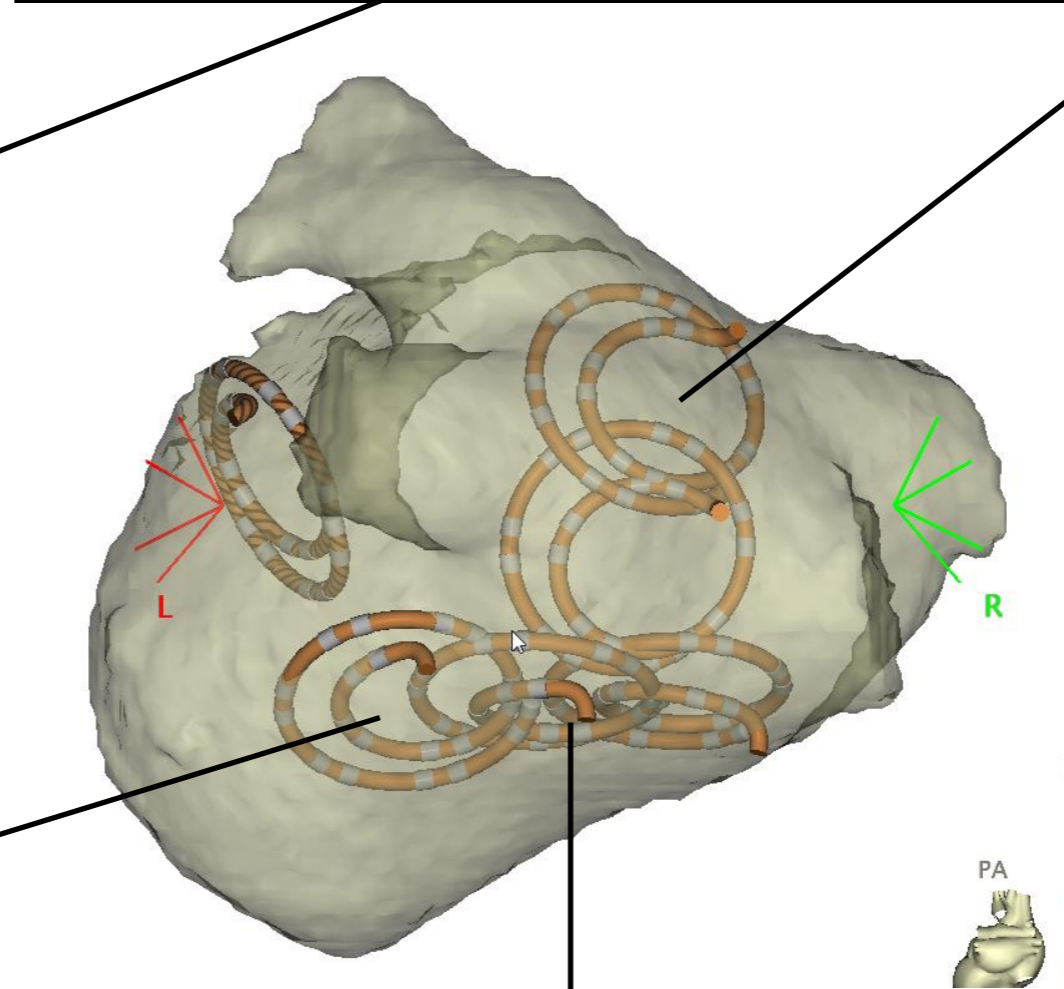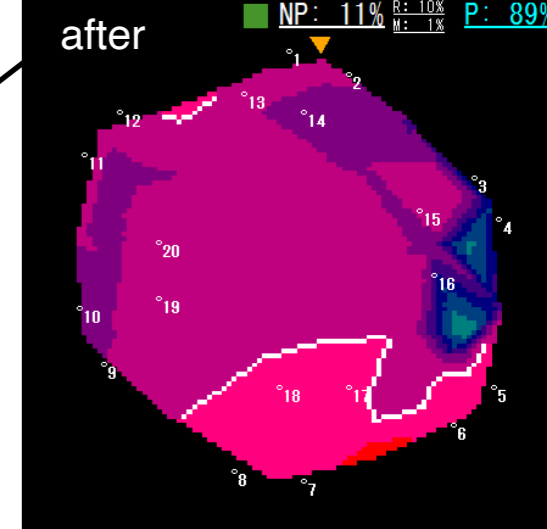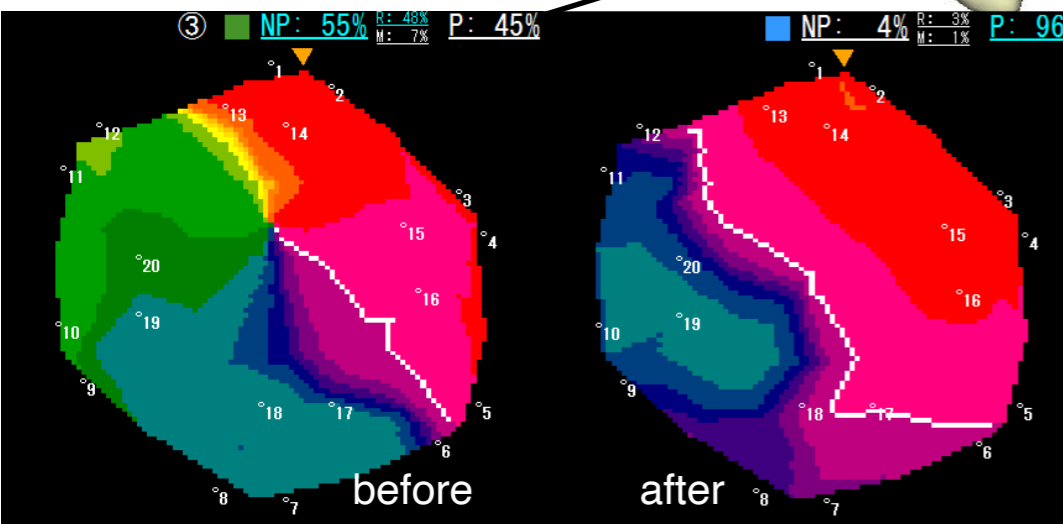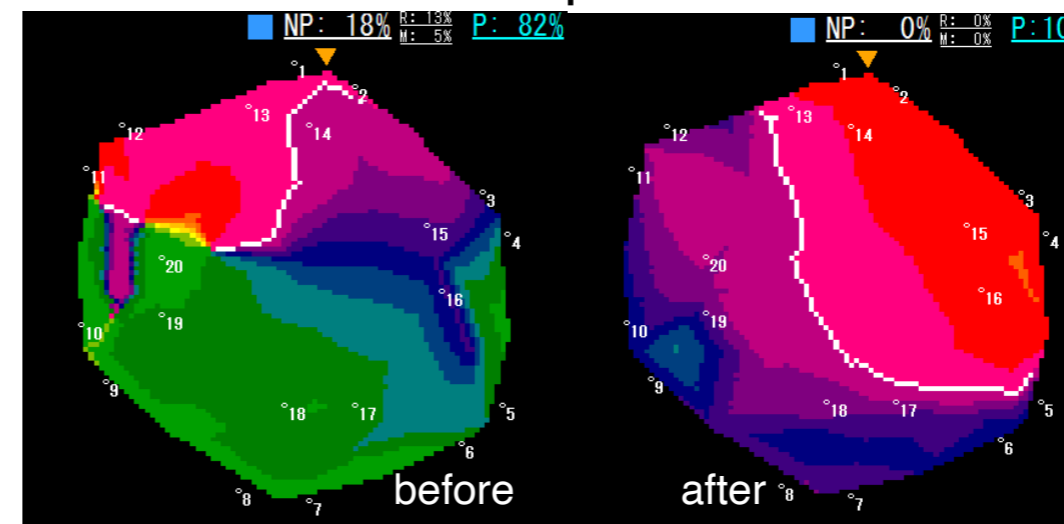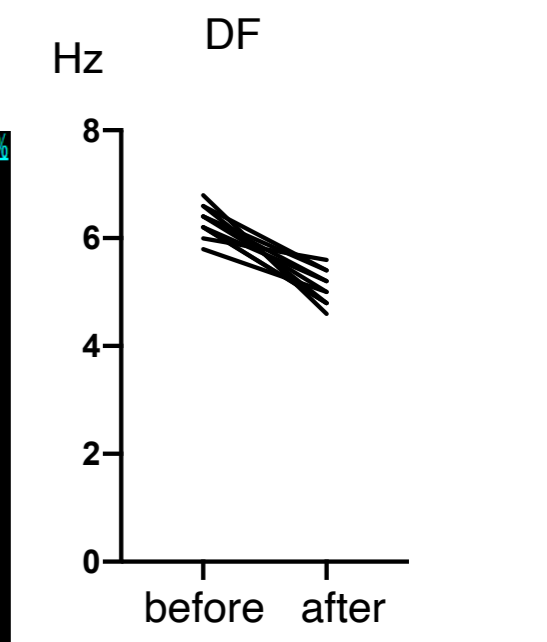

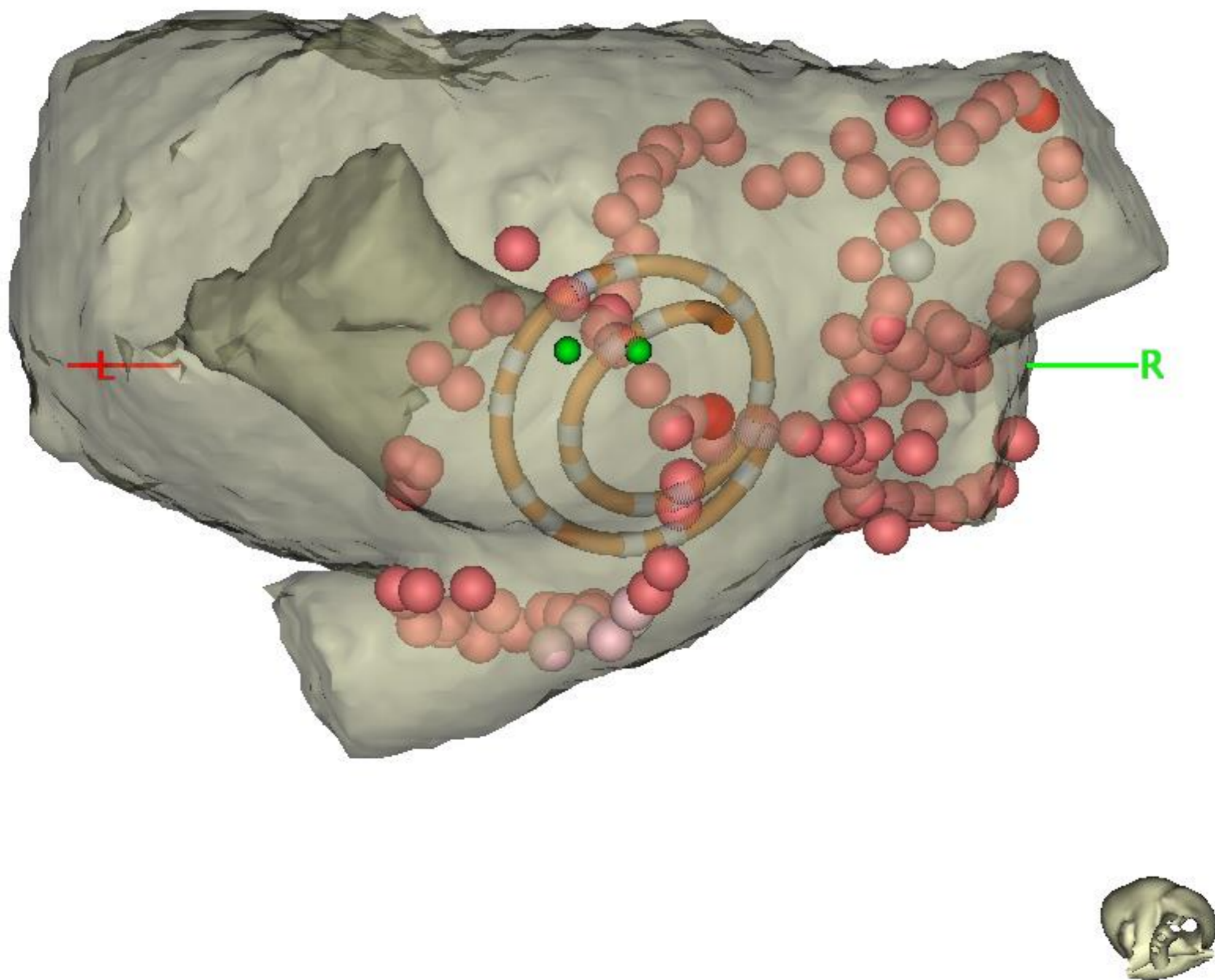

Supplement: Supplementary file 2 — Supplementary Figure 2. [file JOA3-39-327-s001.pdf]

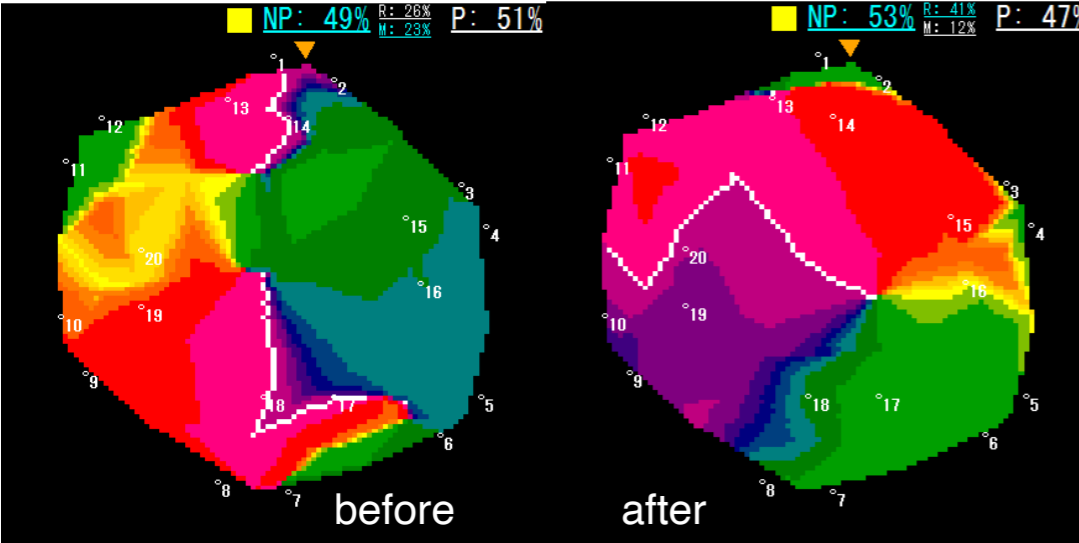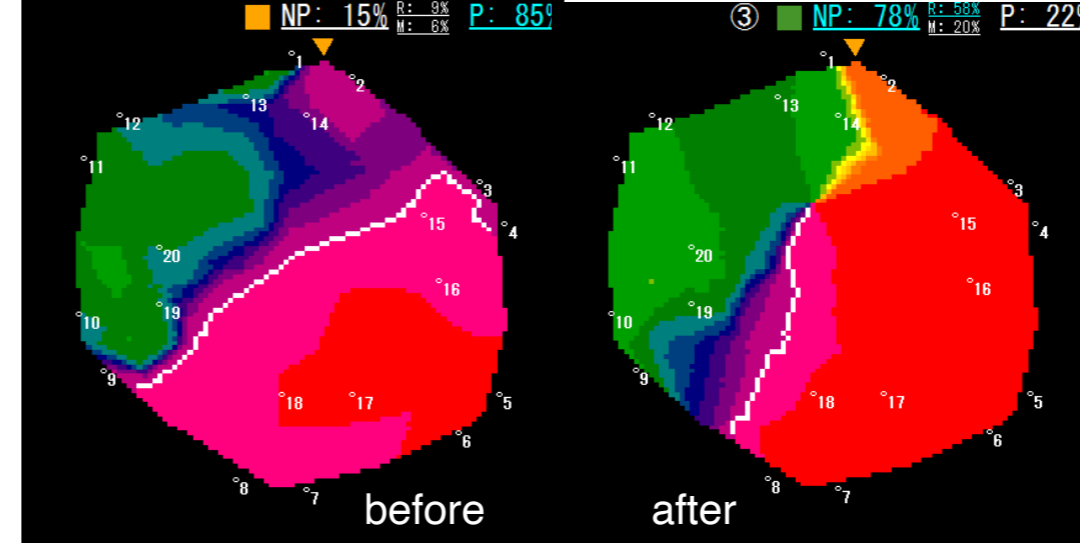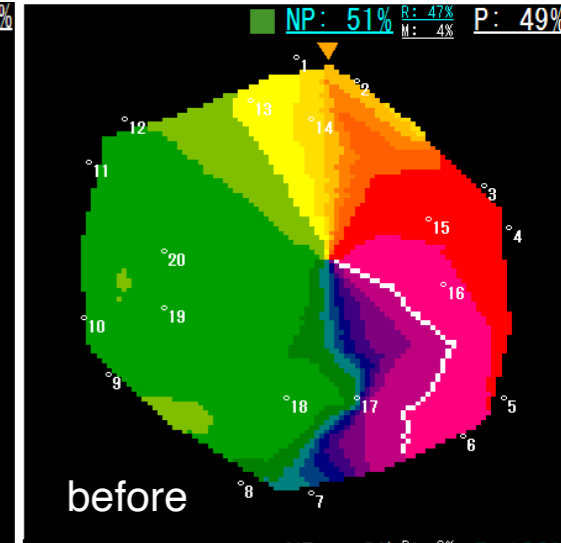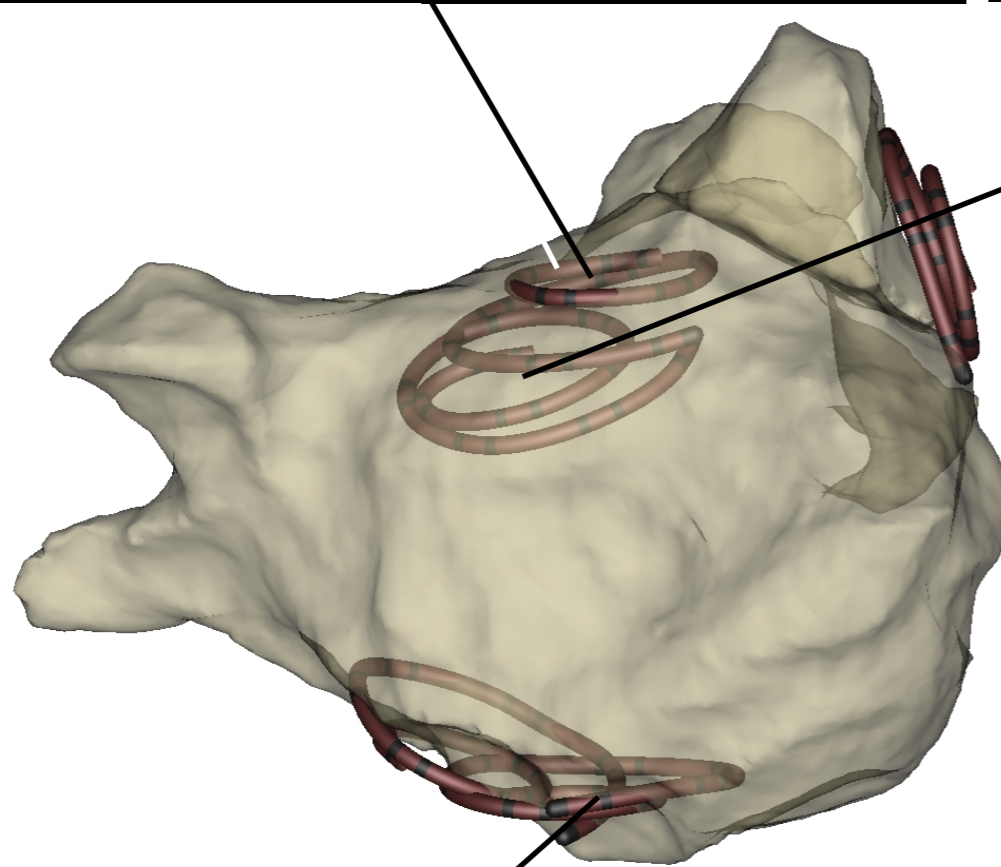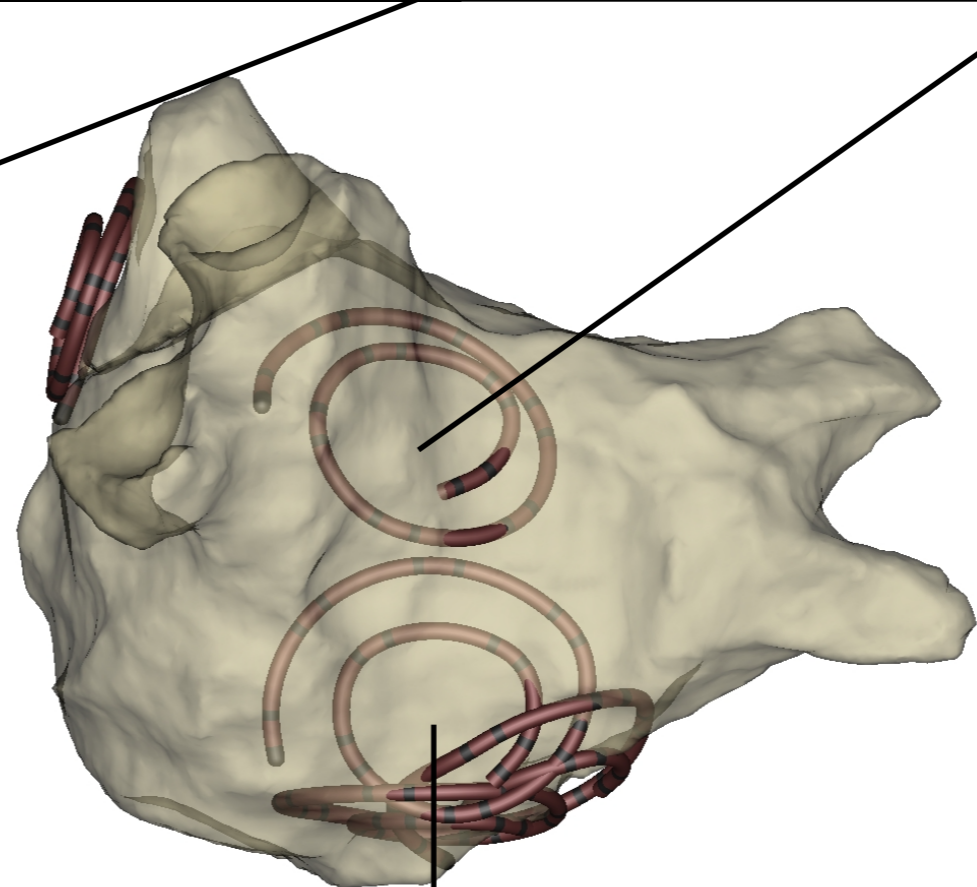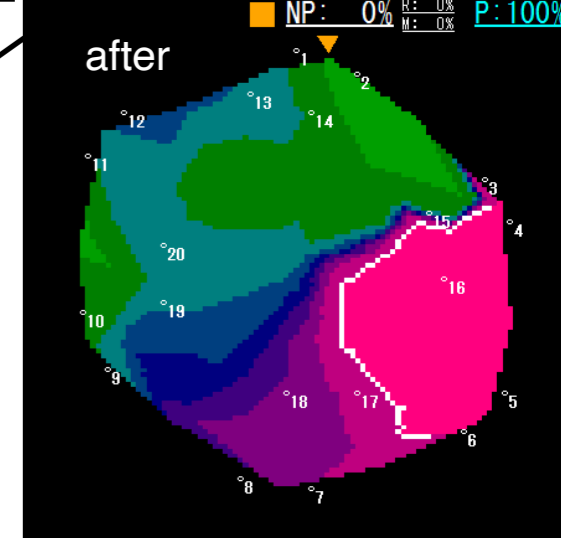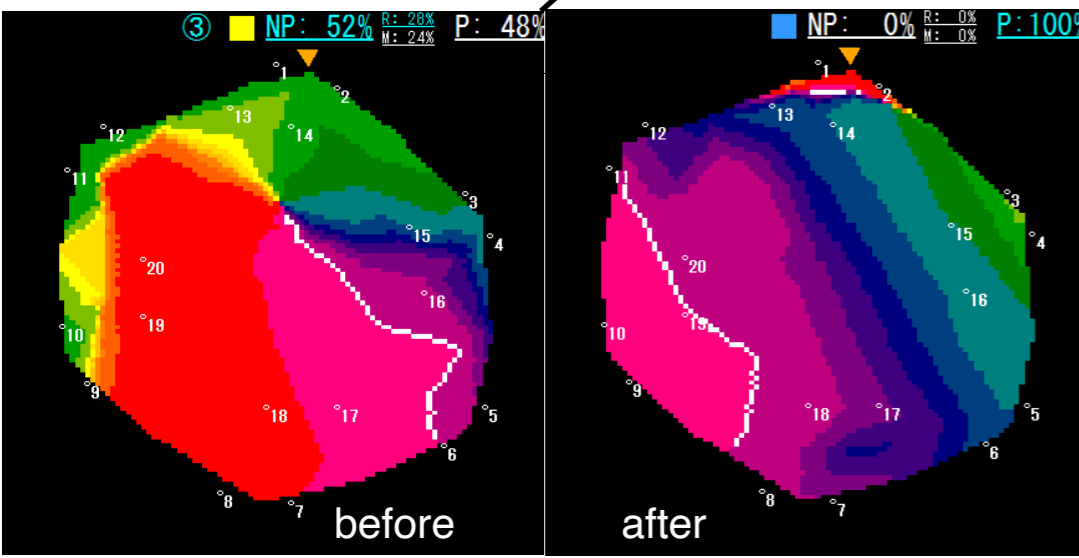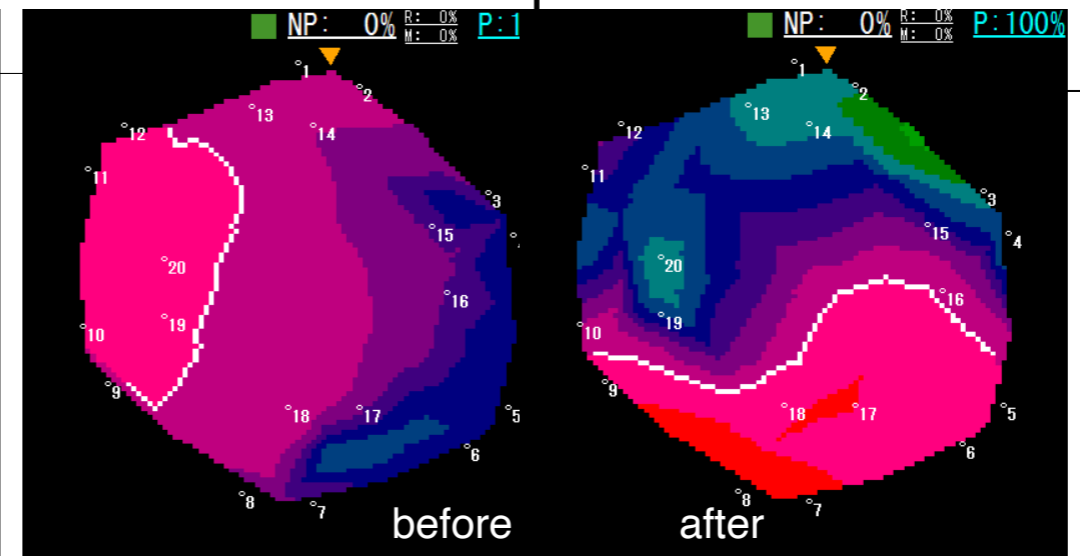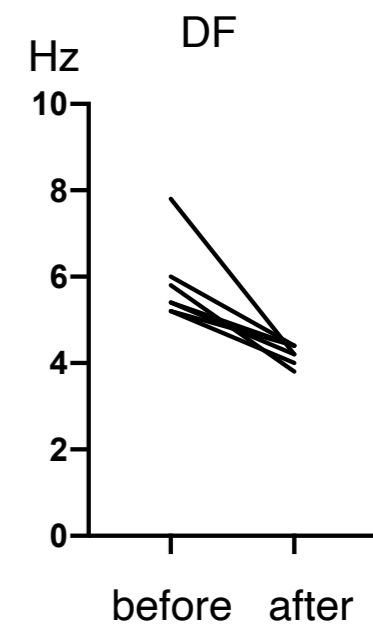

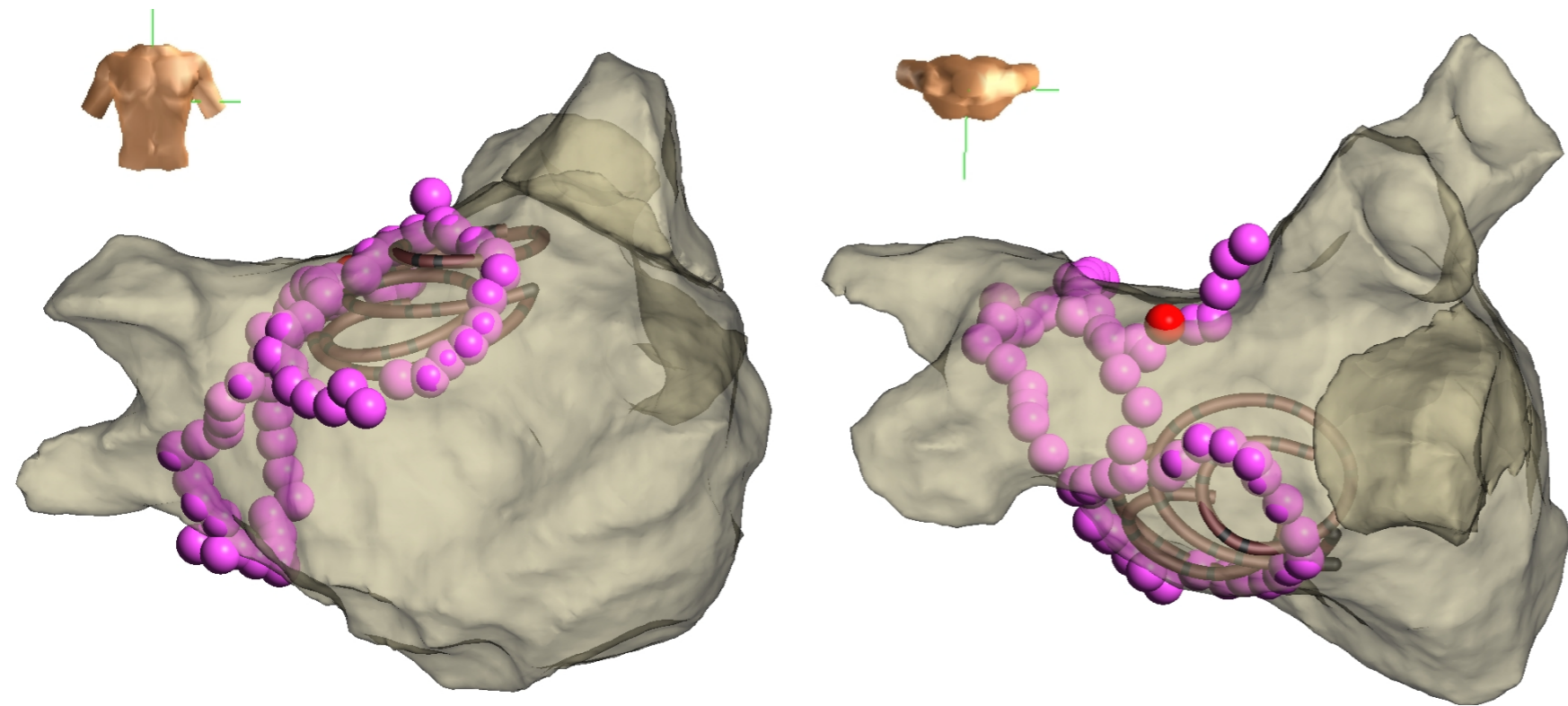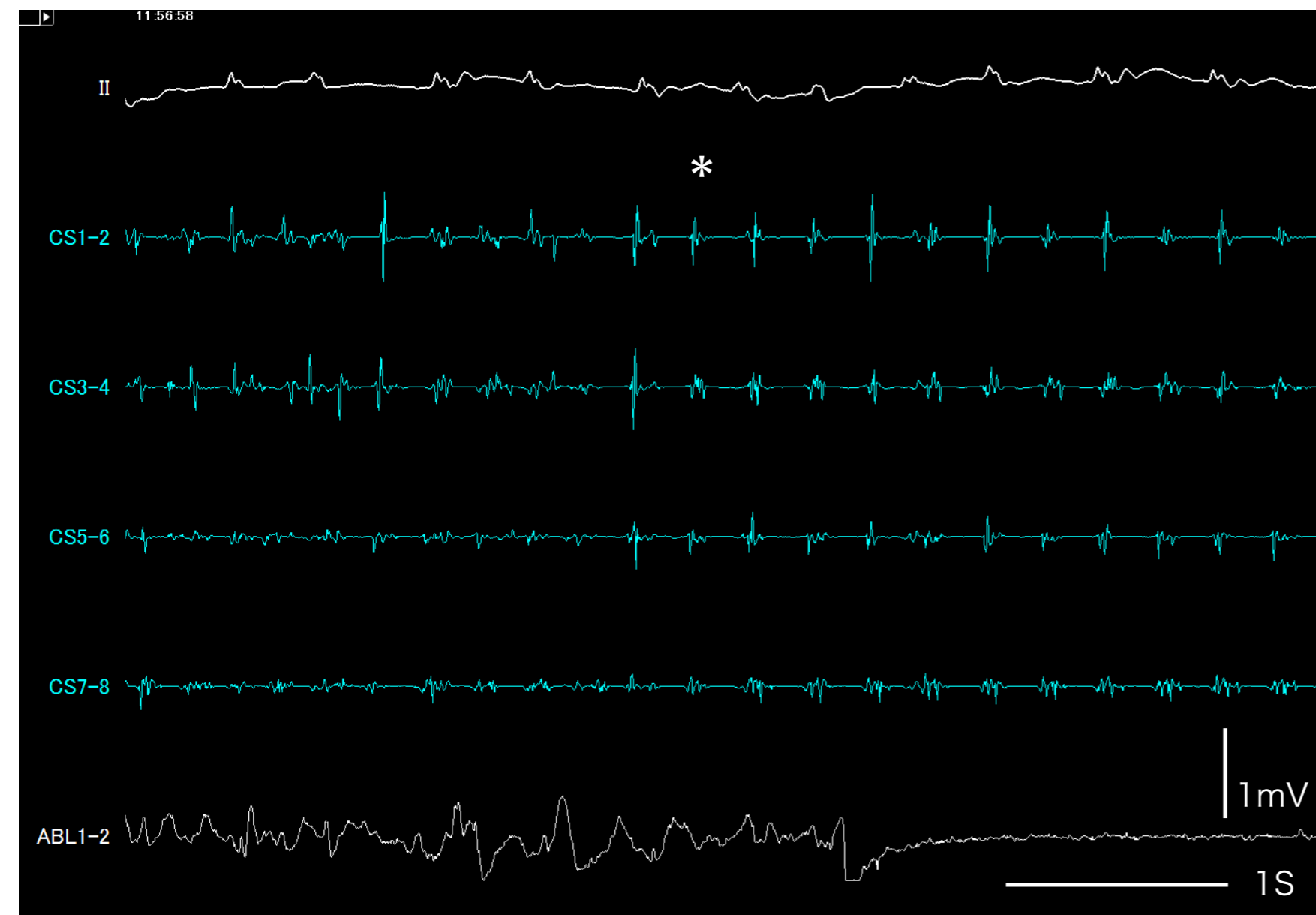

Supplement: Supplementary file 3 — Supplementary Figure 3. [file JOA3-39-327-s005.pdf]
