# Supplementary material for: Organization of atrial fibrillation using a pure sodium channel blocker: Implications of rotor ablation therapy
Source: J Arrhythm. 2023 Mar 31;39(3):327–40. doi: 10.1002/joa3.12844 (PMC10264751; doi:10.1002/joa3.12844)
Supplement: Supplementary file 4 — Supplementary Table 1. [file JOA3-39-327-s002.docx]

Supplementary table 1

Repetitive measurement of %NP value in randomly sampled area

| location | ant | sep | c-bottom | lateral | post | roof | ant | c-bottom | c-bottom | sep | lateral | lateral | roof | post | LIRI | c-bottom | LAA | ant |
| --- | --- | --- | --- | --- | --- | --- | --- | --- | --- | --- | --- | --- | --- | --- | --- | --- | --- | --- |
| first | 20 | 24 | 36 | 27 | 25 | 16 | 77 | 81 | 60 | 14 | 66 | 64 | 26 | 34 | 41 | 26 | 28 | 3 |
| second | 54 | 3 | 37 | 51 | 54 | 41 | 45 | 42 | 37 | 57 | 69 | 55 | 30 | 19 | 30 | 49 | 50 | 58 |
| third | 24 | 39 | 39 | 47 | 36 | 71 | 70 | 43 | 21 | 56 | 27 | 30 | 8 | 36 | 20 | 39 | 26 | 48 |
| fourth | 9 | 32 | 46 | 64 | 44 | 11 | 26 | 60 | 34 | 62 | 14 | 73 | 31 | 38 | 13 | 24 | 38 | 39 |
| fifth | 29 | 33 | 60 | 55 | 65 | 16 | 40 | 43 | 31 | 65 | 11 | 26 | 8 | 30 | 38 | 29 | 40 | 37 |
| sixth | 45 | 32 | 58 | 31 | 40 | 63 | 56 | 28 | 20 | 12 | 56 | 14 | 26 | 27 | 39 | 38 | 45 | 35 |
| seventh | 58 | 8 | 59 | 9 | 17 | 24 | 65 | 27 | 22 | 23 | 25 | 67 | 2 | 29 | 14 | 35 | 62 | 32 |
| eighth | 31 | 23 | 29 | 39 | 21 | 59 | 57 | 59 | 56 | 17 | 36 | 7 | 45 | 20 | 23 | 38 | 37 | 31 |
| ninth | 27 | 34 | 64 | 47 | 24 | 37 | 40 | 41 | 50 | 24 | 48 | 31 | 37 | 21 | 36 | 33 | 66 | 62 |
| tenth | 23 | 35 | 39 | 43 | 60 | 19 | 33 | 73 | 32 | 21 | 40 | 45 | 58 | 39 | 33 | 47 | 19 | 43 |
|  |  |  |  |  |  |  |  |  |  |  |  |  |  |  |  |  |  |  |
| P(Shapiro-Wilk) | 0.411 | 0.051 | 0.182 | 0.78 | 0.477 | 0.168 | 0.84 | 0.375 | 0.217 | 0.02 | 0.675 | 0.586 | 0.71 | 0.34 | 0.234 | 0.728 | 0.806 | 0.384 |

%NP: non-passively activated ratio
