# Supplementary material for: Organization of atrial fibrillation using a pure sodium channel blocker: Implications of rotor ablation therapy
Source: J Arrhythm. 2023 Mar 31;39(3):327–40. doi: 10.1002/joa3.12844 (PMC10264751; doi:10.1002/joa3.12844)
Supplement: Supplementary file 5 — Supplementary Table 2. [file JOA3-39-327-s004.docx]

Supplementary table 2

Highest dominant frequency change with pilsicainide

|  | Organized | Sustained | P value |
| --- | --- | --- | --- |
| roof, n | 6 | 11 |  |
| control, Hz | 7.0±0.8 | 6.6±0.9 | 0.47 |
| pilsicainide. Hz | 5.5±0.9 | 5.4±0.8 | 0.97 |
| posterior | 5 | 9 |  |
| Control, Hz | 6.6±1.1 | 6.6±0.9 | 0.93 |
| pilisicainide, Hz | 5.6±1.1 | 5.6±0.6 | 0.42 |
| low posterior | 13 | 11 |  |
| Control, Hz | 6.2±0.7 | 6.3±0.7 | 0.53 |
| pilisicainide, Hz | 5.0±0.8 | 5.4±0.8 | 0.77 |
| low left PV | 9 | 5 |  |
| Control, Hz | 6.7±0.9 | 6.2±0.9 | 0.78 |
| pilisicainide, Hz | 5.2±1.0 | 5.0±0.3 | 0.31 |
| center inferior | 14 | 14 |  |
| Control, Hz | 6.6±0.8 | 6.1±0.9 | 0.45 |
| pilisicainide, Hz | 5.0±0.8 | 5.2±0.8 | 0.96 |
| left inferior | 13 | 12 |  |
| Control, Hz | 6.4±0.6 | 6.3±0.9 | 0.14 |
| pilisicainide, Hz | 5.4±1.1 | 5.1±0.9 | 0.94 |
| right inferior | 10 | 12 |  |
| Control, Hz | 6.4±0.7 | 6.1±0.8 | 0.13 |
| pilisicainide, Hz | 5.2±0.7 | 5.1±0.7 | 0.99 |
| lateral | 7 | 4 |  |
| Control, Hz | 6.3±0.3 | 6.7±0.7 | 0.21 |
| pilisicainide, Hz | 4.5±0.8 | 5.3±0.8 | 0.66 |
| left atrial appendage | 10 | 13 |  |
| Control, Hz | 6.7±0.8 | 6.6±0.8 | 0.66 |
| pilisicainide, Hz | 5.0±0.7 | 5.2±0.8 | 0.52 |
| anterior | 14 | 13 |  |
| Control, Hz | 6.5±0.7 | 6.5±1.0 | 0.13 |
| pilisicainide, Hz | 4.7±0.7 | 5.1±0.7 | 0.58 |
| septal | 11 | 11 |  |
| Control, Hz | 6.4±0.6 | 6.4±1.0 | 0.06 |
| pilisicainide, Hz | 4.9±0.5 | 4.9±0.8 | 0.01 |
|  |  |  |  |
| maximal DF among areas, n | 14 | 13 |  |
| Control, Hz | 7.2± 0.8 | 6.9±0.8 | 0.87 |
| pilisicainide, Hz | 5.5±1.1 | 5.5±0.8 | 0.7 |
|  |  |  |  |
| minimal DF among areas, n | 14 | 13 |  |
| Control, Hz | 6.1±0.6 | 6.0±0.9 | 0.054 |
| pilisicainide, Hz | 4.5±0.7 | 4.6±0.6 | 0.81 |
|  |  |  |  |
| DF gradient, n | 14 | 13 |  |
| Control, Hz | 1.1±0.9 | 0.9±0.4 | 0.07 |
| pilisicainide, Hz | 1.0±0.9 | 0.9±0.2 | 0.15 |
|  |  |  |  |
| Coefficient of Variation of DF |  |  |  |
| Control | 0.059±0.047 | 0.053±0.021 | 0.11 |
| pilisicainide | 0.071±0.059 | 0.058±0.008 | 0.048 |

DF: dominant frequency
